# MyD88 Adaptor Protein Is Required for Appropriate Hepcidin Induction in Response to Dietary Iron Overload in Mice

**DOI:** 10.3389/fphys.2018.00159

**Published:** 2018-03-05

**Authors:** Antonio Layoun, Macha Samba-Mondonga, Gabriela Fragoso, Annie Calvé, Manuela M. Santos

**Affiliations:** ^1^Nutrition and Microbiome Laboratory, Centre de Recherche du Centre Hospitalier de l'Université de Montréal, Montréal, QC, Canada; ^2^Département de Médecine, Université de Montréal, Montréal, QC, Canada

**Keywords:** iron, hepcidin, MyD88, Smad4, hemochromatosis, Bmp6, Bmp2

## Abstract

Iron homeostasis is tightly regulated to provide virtually all cells in the body, particularly red blood cells, with this essential element while defending against its toxicity. The peptide hormone hepcidin is central to the control of the amount of iron absorbed from the diet and iron recycling from macrophages. Previously, we have shown that hepcidin induction in macrophages following Toll-like receptor (TLR) stimulation depends on the presence of myeloid differentiation primary response gene 88 (MyD88). In this study, we analyzed the regulation of iron metabolism in *MyD88*^−/−^ mice to further investigate MyD88 involvement in iron sensing and hepcidin induction. We show that mice lacking MyD88 accumulate significantly more iron in their livers than wild-type counterparts in response to dietary iron loading as they are unable to appropriately control hepcidin levels. The defect was associated with inappropriately low levels of Smad4 protein and Smad1/5/8 phosphorylation in liver samples found in the *MyD88*^−/−^ mice compared to wild-type mice. In conclusion, our results reveal a previously unknown link between MyD88 and iron homeostasis, and provide new insights into the regulation of hepcidin through the iron-sensing pathway.

## Introduction

Iron homeostasis in mammals is tightly regulated to meet body requirements while preventing iron toxicity, as iron can participate in the generation of harmful free radicals (Hentze et al., 2004; Sheftel et al., 2011). Most of this regulation takes place at the level of intestinal iron absorption, which is negatively controlled by levels of the peptide hormone hepcidin, encoded by the *HAMP* gene (Ganz and Nemeth, 2012). Hepcidin also plays a major role in regulating iron release from macrophages. These cells are responsible for iron recycling, with high hepcidin levels ultimately leading to iron accumulation in macrophages (Ganz and Nemeth, 2012). Erythropoietic activity, hypoxia, host defense, and multiple signals reflecting systemic iron stores and circulating levels converge to regulate hepcidin production, mostly in the liver, and affect body iron homeostasis (Huang et al., 2009).

Two major pathways that contribute to hepcidin regulation are the inflammatory pathway mediated through IL-6/STAT3 signaling (Nemeth et al., 2004; Verga Falzacappa et al., 2007), and the iron-sensing pathway, which is mediated through BMP/SMAD signaling (Rishi et al., 2015). BMP/SMAD signaling is modulated in response to body iron status: increased dietary iron levels stimulate the production of bone morphogenetic protein 6 (BMP6) (Kautz et al., 2008; Meynard et al., 2009; Corradini et al., 2011), which then binds to heteromeric complexes containing type II and type I BMP receptors (BMPRI/II) (Parrow and Fleming, 2014). The binding of BMP6 to its receptors results in the recruitment and subsequent phosphorylation of SMADs 1, 5, and 8 (SMAD1/5/8). In turn, SMAD1/5/8 binds to SMAD4 to form a transcriptional complex that translocates to the nucleus and binds to specific DNA elements in the hepcidin promoter (Casanovas et al., 2009). While BMP6 is a key regulator of hepcidin expression and systemic iron homeostasis (Andriopoulos et al., 2009; Meynard et al., 2009), more recent work identified BMP2 as another BMP ligand indispensable for iron homeostasis *in vivo* that is non-redundant with BMP6 (Canali et al., 2017; Koch et al., 2017).

Hepcidin regulation through IL-6/STAT3 signaling occurs during inflammation or infection, when the host's innate immune response activates a sequence of events that limits iron availability by sequestering iron and down-regulating intestinal iron absorption (Weiss, 2009). Production of cytokines, particularly IL-6, leads to STAT3 phosphorylation, translocation into the nucleus, and binding to STAT3-recognizing DNA elements located in the hepcidin promoter (Verga Falzacappa et al., 2007).

Previously, we have shown that hepcidin induction in macrophages following stimulation of Toll-like receptors (TLRs) depends on the presence of myeloid differentiation primary response gene 88 (MyD88) (Layoun and Santos, 2012). In addition to macrophages, TLRs and MyD88 are also expressed in hepatocytes (Liu et al., 2002) and it has been demonstrated that LPS stimulation induces hepcidin expression in hepatocytes via a MyD88-dependent signaling pathway (Lee et al., 2017).

Almost all TLRs use MyD88 as a universal adapter protein to activate the transcription factor NF-κB and cytokine production through the common MyD88-dependent signaling pathway. TRIF is another adapter protein used by TLR3 and TLR4 to activate NF-κB through the TRIF-dependent signaling pathway (Yamamoto et al., 2003). Both MyD88 and Trif-deficient mice have impaired production of inflammatory cytokines (Takeuchi and Akira, 2010). In previous work, we showed that MyD88-deficient mice are unable to sustain an acute hypoferremic response triggered by lipopolysaccharide (LPS), a TLR4 ligand (Layoun et al., 2012). The contribution of TLRs/MyD88 signaling for hepcidin expression through the inflammatory pathway has been further demonstrated using a variety of cellular and animal models (Wang et al., 2009; Xiong et al., 2016; Lee et al., 2017).

Since both the iron-sensing pathway and inflammatory pathways reveal overlap in hepcidin induction when converging at SMAD1/5/8 phosphorylation and SMAD4 binding, we investigated the potential role of MyD88 in iron sensing by analyzing iron metabolism in MyD88-deficient mice (*MyD88*^−/−^).

## Materials and methods

### Animals

This study was carried out in accordance with Canadian Council on Animal Care guidelines. The protocol was approved by the institutional Animal Care Committee of the CRCHUM.

C57BL/6 (B6) wild-type (Wt) and Trif-deficient mice (C57BL/6JTicam1^Lps2^/J or Trif^LPS2/LPS2^, B6 background), were purchased from Jackson Laboratories (Bar Harbor, ME). *MyD88*^−/−^ mice in the B6 genetic background were kindly provided by Dr. Shizuo Akira (Research Institute for Microbial Diseases, Osaka University, and Japan Science and Technology Agency, Tokyo, Japan) and were maintained as described previously (Adachi et al., 1998). Mice were maintained under standard 12:12 h light/dark conditions at the Centre de recherche du CHUM (CRCHUM). The animals used in the experiments were female and were permanently housed under specific pathogen-free conditions.

### Animal treatments

Mice were fed a commercial diet containing ~200 mg of iron per kg (Teklad Global 18% protein rodent diet; Harlan Teklad, Madison, WI). Dietary iron overload was produced by feeding 8-week-old mice the same commercial diet supplemented with 25 g carbonyl iron per kg (2.5% wt/wt carbonyl iron; Sigma-Aldrich, St. Louis, MO, USA) for 2 weeks (Jiang et al., 2008). All animals were 10-weeks old at the end of the experiments.

### Hematologic measurements and transferrin saturation

Red blood cell (RBC) count, hemoglobin (Hb), hematocrit (HCT), and mean corpuscular volume (MCV) were measured with an automated cell counter calibrated for murine samples (ABC vet counter; ABX Hématologie, Montpellier, France). Serum iron was assessed by colorimetric assay with the Kodak Ektachem DT60 system (Johnson & Johnson, Ortho Clinical Diagnostics, Mississauga, ON).

### Measurement of tissue iron concentration

Spleen, and liver iron concentrations (LIC) were assessed by acid digestion of tissue samples, followed by iron quantification with atomic absorption spectroscopy (Wienk et al., 1997).

### Serum ferritin assay

Ferritin was measured in serum with an enzyme-linked immunosorbent assay (ELISA) kit as per manufacturer's instructions (mouse Ferritin ELISA kit, Kamiya Biomedical, Seattle).

### Histology

Liver tissue sections were stained with Perls' Prussian blue for ferric iron detection (iron stain kit; Sigma Immunochemicals).

### Quantitative RT-PCR

Total RNA was isolated with Trizol reagent (Invitrogen, Burlington, ON), and reverse transcription was performed with the Omniscript RT kit (QIAGEN, Mississauga, ON). mRNA expression levels were measured by real-time PCR in a Rotor Gene 3000 Real Time DNA Detection System (Montreal Biotech, Kirkland, QC) with QuantiTect SYBRGreen I PCR kits (QIAGEN, Mississauga, ON) as described (Makui et al., 2005). The primers used in this study are presented in Table 1. Expression levels were normalized to the housekeeping gene β-actin (*Actb*).

**Table 1:** Primers used for qPCR analysis of mRNA levels.

**Gene**		**Sequence**
*Actb*	Forward	TGTTACCAACTGGGACGACA
	Reverse	GGTGTTGAAGGTCTCAAA
*Hamp*	Forward	CCTATCTCCATCAACAGATG
	Reverse	AACAGATACCACACTGGGAA
*Atoh8*	Forward	CACCATCAGCGCAGCCTTC
	Reverse	AATCCAGCAGGTCAGCAAAG
*Id1*	Forward	ACCCTGAACGGCGAGATCA
	Reverse	TCGTCGGCTGGAACACATG
*Bmp6*	Forward	GACAAGGAGTTCTCCCCACA
	Reverse	CCAGCCAACCTTCTTCTGAG
*Bmp2*	Forward	GCCTGCACCCTGTTCTCTGA
	Reverse	ATGTTCAAACACATATCCCTGGAA

### SDS–PAGE and western blot analysis

Livers were removed, rinsed in ice-cold PBS, and used to prepare liver nuclear extracts with Nuclear Extract Kits (Active Motif, Carlsbad, CA). Total cell lysates and, when indicated, nuclear protein extracts were separated by 10% SDS–PAGE and blotted onto nitrocellulose membranes (Bio-Rad Laboratories, Mississauga, Ontario). The membranes were immunoblotted with the following antibodies: ferritin (FTH1) (1:1000) (Alpha Diagnostic International, San Antonio, TX), phospho-Smad5 (1:1000) (Abcam, Cambridge, MA), Smad1 (1:1000) (Cell Signaling, Danvers, MA), Smad4 (1:1000) (Santa Cruz Biotechnology, Santa Cruz, CA), phospho-Stat3 (1:1000) (Cell Signaling, Danvers, MA), Stat3 (1:1000) (Cell Signaling, Danvers, MA), HDAC1 (1:1000) (Santa Cruz, Biotechnology, Santa Cruz, CA), β-Tubulin (1:1000) (Cell Signaling, Danvers, MA), and β-actin (1:10000) (Abcam, Cambridge, MA). Anti–rabbit IgG (1:5000) (Cell Signaling, Danvers, MA) or anti–mouse IgG (1:5000) (GE Healthcare, Amersham Biosciences, Baie d'Urfe, QC, Canada) was used as secondary antibody. Antigen-antibody complexes were visualized with the ECL Western Blotting Detection Reagent (GE Healthcare).

### Statistical analysis

All statistics were calculated with Prism software (GraphPad, San Diego, CA), with a pre-specified significant *P*-value of 0.05. Multiple comparisons were evaluated statistically by one-way analysis of variance (ANOVA) followed by the Bonferroni multiple comparison test.

## Results

### Increased liver iron stores in *MyD88*^−/−^ mice

**Figure 1:**
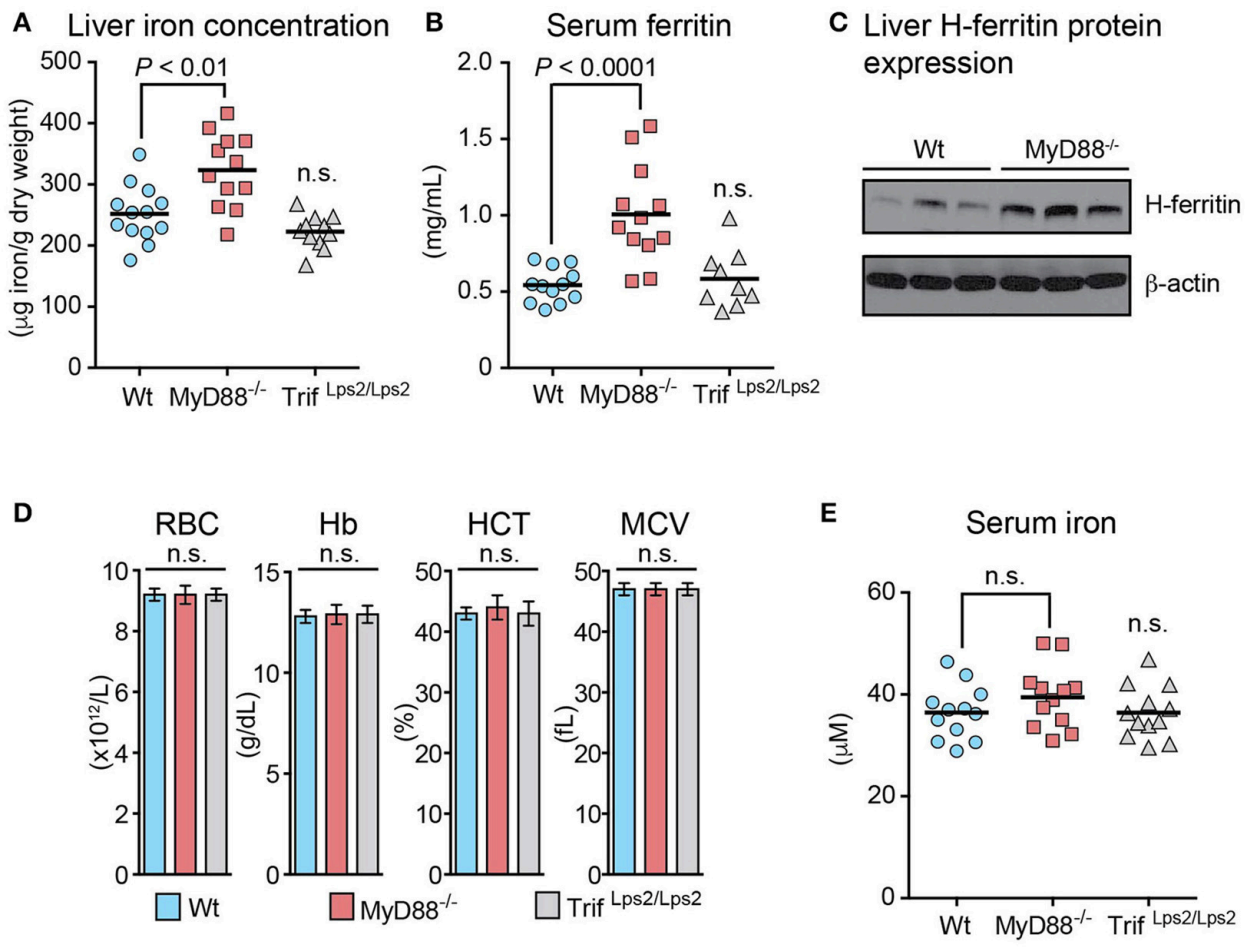
Increased liver iron stores with normal erythropoietic parameters in MyD88-deficient mice. Wild-type (Wt), MyD88-deficient (MyD88^−/−^) and Trif-deficient (Trif^Lps2/Lps2^) mice were fed a standard diet. **(A)** Liver iron concentration measured by atomic absorption spectroscopy. **(B)** Serum ferritin levels. **(C)** Representative western blot showing H-ferritin levels in the liver of Wt and *MyD88*^−/−^ mice. The blot was stripped and reprobed with an antibody to β-actin as loading control. **(D)** Erythroid parameters: red blood cells (RBC), hemoglobin (Hb), hematocrit (HCT), and mean corpuscular volume (MCV). Results are presented as mean ± SEM. **(E)** Serum iron. Results are presented as combined data from two independent experiments performed with *n* ≥ 5 mice per group. Statistical analysis was performed with one-way ANOVA. n.s., not significant compared to Wt mice.

We have previously reported that mice deficient in MyD88, unlike Trif-deficient mice, are unable to maintain LPS-induced, acute hypoferremic response as they fail to divert iron from the circulation into the spleen (Layoun et al., 2012). To further investigate the role of MyD88 in iron metabolism, we first examined MyD88-deficient mice and assessed iron levels in the liver, the major iron storage organ in the body. Compared to Wt and Trif-deficient mice that were used as controls, *MyD88*^−/−^ animals presented consistently higher iron concentrations in the liver (Figure 1A). In addition, *MyD88*^−/−^ mice had approximately twice the level of serum ferritin (Figure 1B) and higher levels of hepatic H-ferritin protein (Figure 1C) than the levels observed in Wt mice, further confirming that MyD88 deficiency results in elevated liver iron storage. The increased iron concentration in *MyD88*^−/−^ livers was not due to altered erythropoiesis or circulating iron since the erythroid parameters and serum iron levels in *MyD88*^−/−^ mice were similar to those in Wt and Trif-deficient mice (Figures 1D,E).

### *MyD88*^−/−^ mice exhibit inability to appropriately regulate hepcidin in response to dietary iron loading

**Figure 2:**
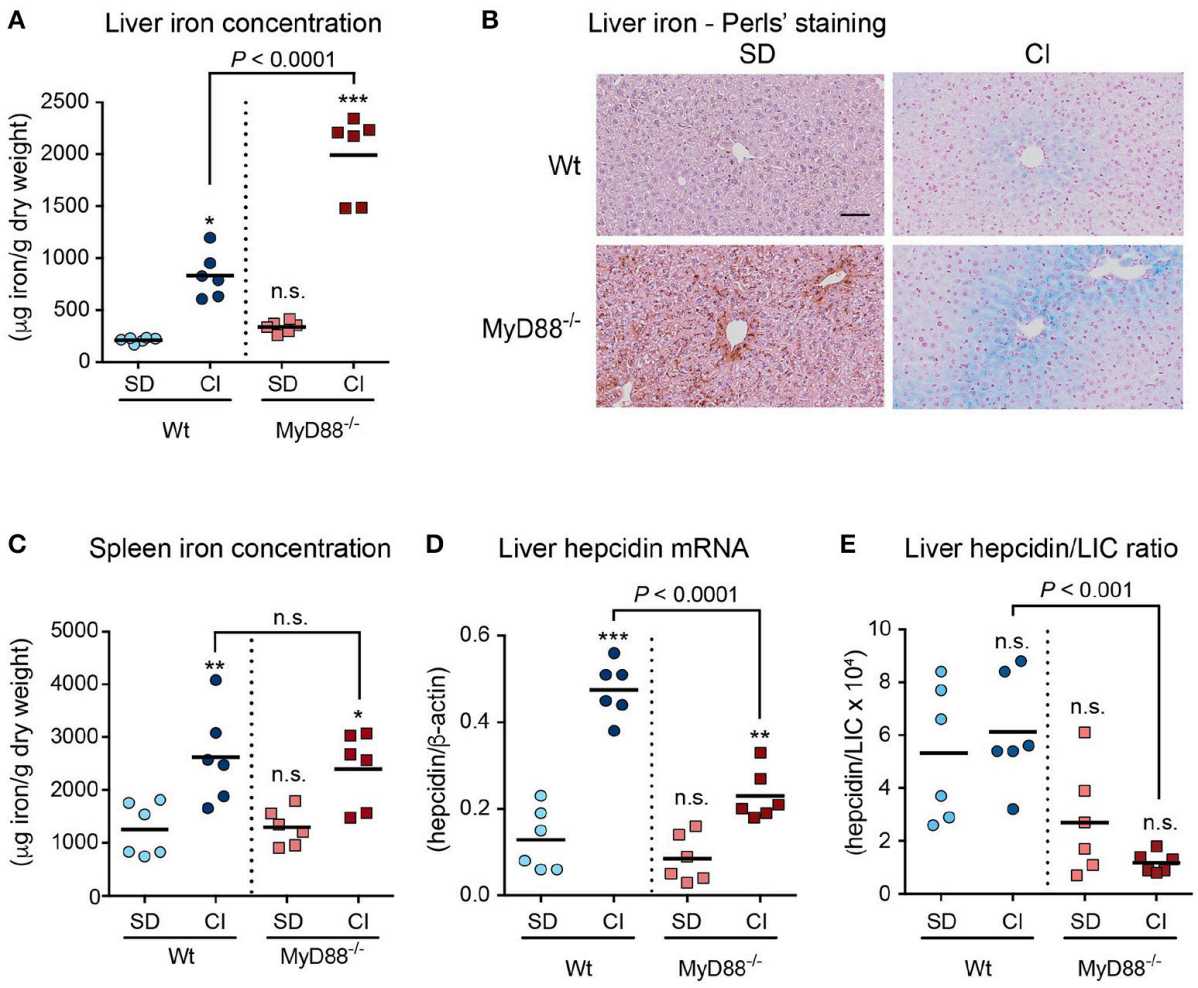
Inability to appropriately upregulate hepcidin in response to iron challenge in MyD88-deficient mice. Wild-type (Wt) and MyD88-deficient (MyD88^−/−^) mice were fed a standard diet (SD) or carbonyl iron supplemented diet (CI) for 2 weeks. **(A)** Liver iron concentration measured by atomic absorption spectroscopy. **(B)** Ferric iron staining in liver sections. Left panels: Basal iron levels in the liver of mice fed SD, detected by DAB-enhanced Perl's staining (Smith et al., 1997) (brown) with hematoxylin (blue) and eosin counterstaining. Right panels: Iron levels in the liver of mice fed CI, detected by Perls' Prussian blue staining. Scale bar = 50 μm. **(C)** Spleen iron concentration. **(D)** Liver hepcidin mRNA levels. **(E)** Ratio hepcidin/liver iron concentration (LIC). Results are representative of a minimum of three independent experiments using *n* = 4–6 mice per group in each experiment. Statistical analysis was performed with one-way ANOVA. ^*^P < 0.01, ^**^P < 0.001, and ^***^P < 0.0001, compared with mice fed SD; n.s., not significant.

Next, we investigated whether MyD88 deficiency might impact the regulation of hepcidin with consequent storage of excess iron in the liver. Mice were challenged with an iron-enriched diet for 2 weeks to mimic chronic iron overload. This treatment resulted in a 5.6-fold increase of iron accumulation in the livers of *MyD88*^−/−^ mice compared to the 3.5-fold increase observed in Wt mice (Figure 2A), and was further confirmed by Perls' staining for ferric iron in liver samples (Figure 2B). In contrast, iron levels in the spleen of iron-loaded *MyD88*^−/−^ mice were similar to Wt mice (Figure 2C), indicating an inability to proportionately accumulate iron in this organ. These results suggest that *MyD88*^−/−^ mice may have a defect in adjusting the levels of the iron regulatory hormone hepcidin (Ganz and Nemeth, 2012); therefore, we next assessed mRNA expression levels of liver hepcidin. Despite showing increased hepcidin mRNA expression upon chronic dietary iron-loading, *MyD88*^−/−^ mice have comparatively lower levels of hepcidin than iron-loaded Wt mice (Figures 2D,E).

Altogether, our data suggest that mice lacking functional MyD88 adaptor molecules present insufficient hepcidin induction in response to dietary iron loading.

### Defective signaling through the BMP/SMAD pathway in *MyD88*^−/−^mice

**Figure 3:**
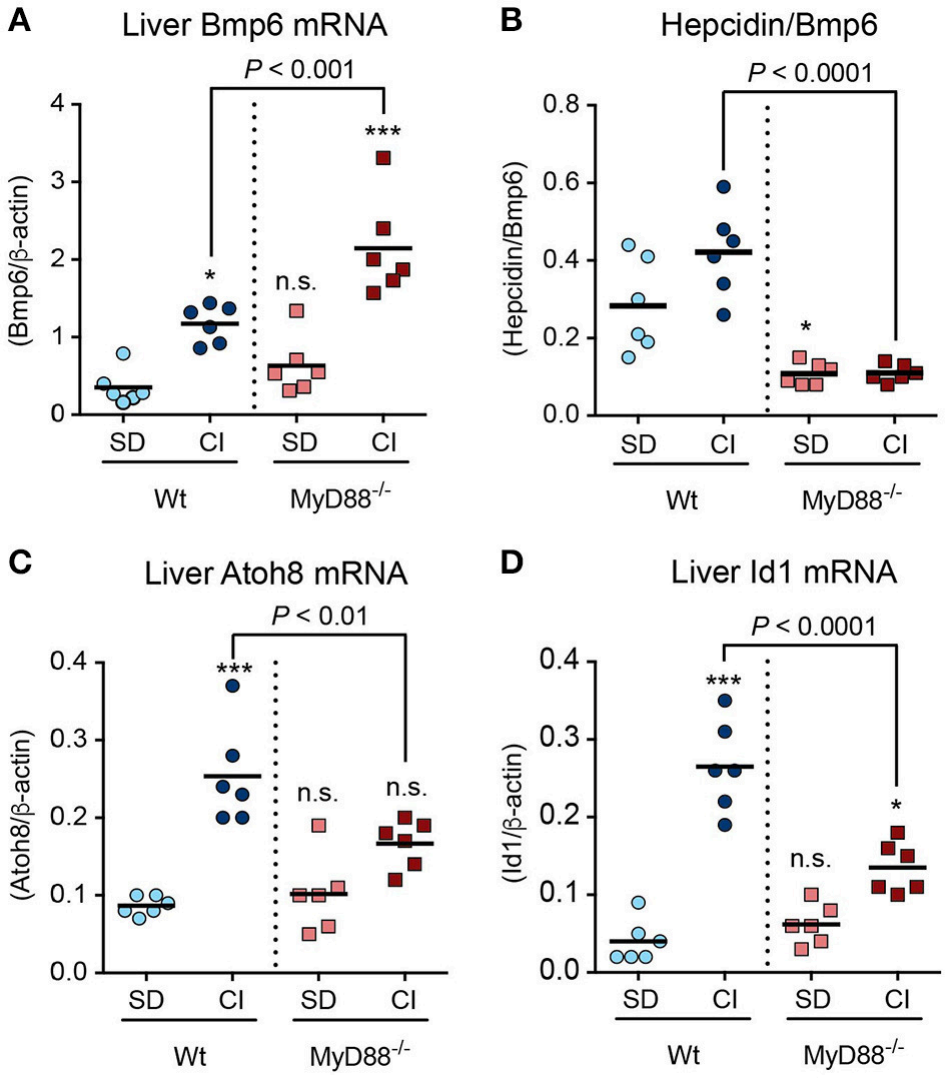
Defective signaling though the BMP6/SMAD pathway in response to dietary iron-loading in *MyD88*^−/−^ mice. Wt and MyD88^−/−^ mice were fed a standard diet (SD) or carbonyl iron supplemented diet (CI) for 2 weeks. **(A)** *Bmp6* mRNA levels in the liver. **(B)** Hepcidin/Bmp6 mRNA ratio. **(C)** *Atoh8* mRNA levels in the liver. **(D)** *Id1* mRNA levels in the liver. The results are representative of a minimum of three independent experiments using *n* = 4–6 mice per group in each experiment. Statistical analysis was performed with one-way ANOVA: ^*^P < 0.01, ^***^P < 0.0001 and n.s., not significant compared with mice fed SD.

*In vivo*, iron loading induces the expression of *Bmp6* mRNA in the liver (Kautz et al., 2008), which is believed to be an initial step in the activation of the BMP/SMAD1/5/8 signaling cascade for hepcidin induction in response to iron overloading (Meynard et al., 2009). Thus, we next sought to understand whether BMP6 activation was compromised in *MyD88*^−/−^ mice. Liver *Bmp6* mRNA expression was induced by iron in both Wt and *MyD88*^−/−^ mice, and was even higher in iron-loaded *MyD88*^−/−^ mice, demonstrating that the absence of MyD88 did not affect the induction of *Bmp6* (Figure 3A). Since BMP2 has also been shown to be necessary for iron homeostasis *in vivo* (Canali et al., 2017; Koch et al., 2017), we also measured *Bmp2* mRNA expression in the liver. We found that *Bmp2* mRNA levels were induced by dietary iron to similar levels in both Wt and *MyD88*^−/−^ mice (Supplementary Figure 1). However, induction of BMP ligands was not accompanied by the expected rise in hepcidin. Consequently, the ratio between *Hamp* mRNA and *Bmp6* mRNA was persistently lower in *MyD88*^−/−^ mice compared to Wt mice (Figure 3B), and indicated a defect in the downstream signaling transmission initiated by Bmp6. Measurements of the mRNA levels of *Atoh8* and *Id1*, two genes shown to be induced by iron through the BMP6/SMAD4 pathway (Kautz et al., 2008), further reinforced the idea that the main defect in *MyD88*^−/−^ mice resides in the BMP6/SMAD4 down-stream signaling pathway of targeted genes. In fact, the mRNA levels of both genes were significantly lower in *MyD88*^−/−^ mice compared to wild-type mice in response to dietary iron loading (Figures 3C,D).

**Figure 4:**
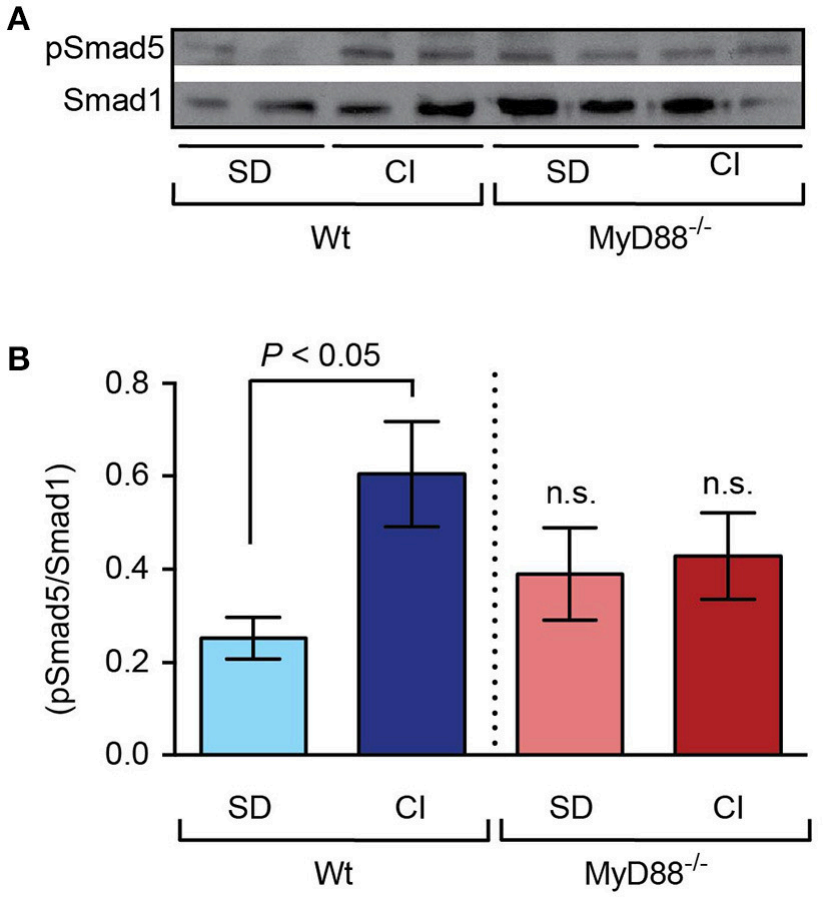
Lack of Smad5 phosphorylation in response to dietary iron-loading in *MyD88*^−/−^ mice. Liver nuclear extracts from mice fed standard diet (SD) and carbonyl iron supplemented diet (CI) were analyzed by western blots. **(A)** One representative blot is presented that was probed with antibodies against phosphorylated Smad5 (pSmad5) and total Smad1. **(B)** Graphic depicting densitometric quantification of western blots from three independent experiments. Statistical analysis was performed with one-way ANOVA: n.s., not significant compared with Wt mice fed the same diet.

BMP6 signals through the phosphorylation of SMAD proteins 1, 5, and 8 (Kersten et al., 2005), which translocate into the nucleus where they regulate the transcription of specific target genes, including hepcidin (Pantopoulos et al., 2012). Hence, we next analyzed phosphorylation levels of Smad5 in liver nuclear extracts from dietary iron-loaded *MyD88*^−/−^ and Wt mice. Dietary iron loading induced phosphorylation of liver Smad5 proteins in Wt mice, as expected (Besson-Fournier et al., 2017; Canali et al., 2017). In contrast, despite higher hepatic iron levels found in *MyD88*^−/−^ mice, no significant differences regarding Smad5 phosphorylation were detected in this mouse strain when comparing the standard diet with the carbonyl iron supplemented diet (Figures 4A,B).

**Figure 5:**
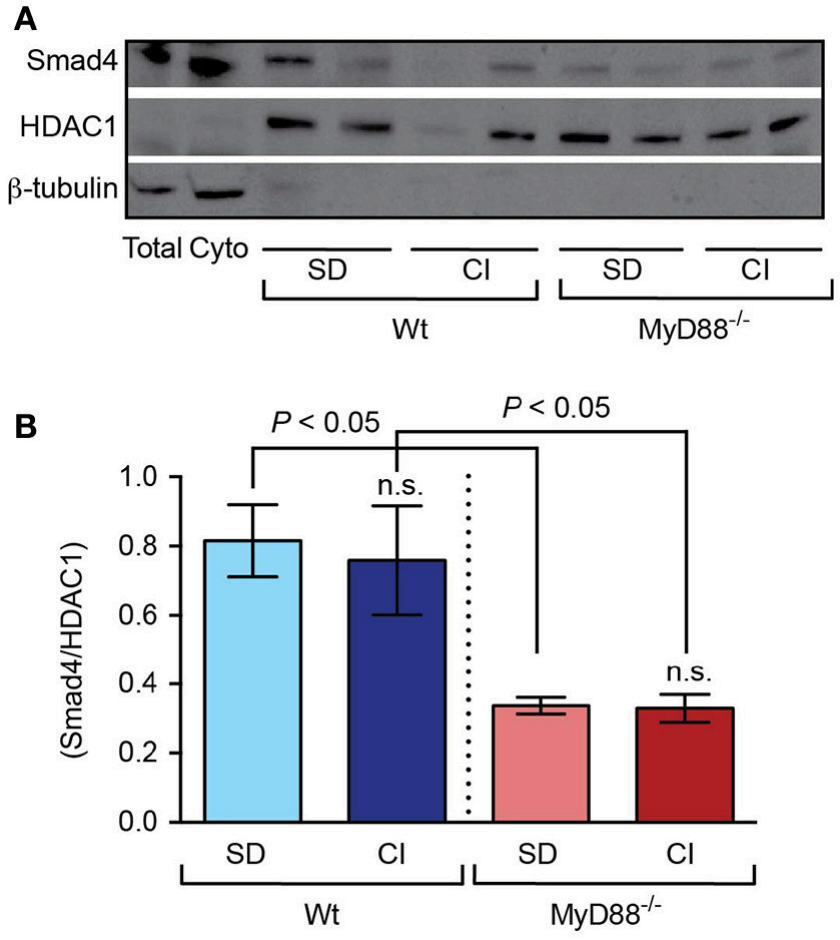
Defective signaling in response to dietary iron-loading in *MyD88*^−/−^ mice involves diminished Smad4 protein in the liver. Liver nuclear extracts from mice fed standard diet (SD) and carbonyl iron supplemented diet (CI) were analyzed by western blots. **(A)** One representative blot probed with an antibody against Smad4. Blots were stripped and reprobed with an antibody to HDAC1 (nuclear marker) and to β-tubulin (cytoplasmic marker). Total, liver total extract; Cyt, liver cytoplasmic extract. **(B)** Graphic depicting densitometric quantification of western blots from three independent experiments. Statistical analysis was performed with one-way ANOVA: n.s., not significant compared with mice fed SD.

For the activation signal to be successfully transmitted, phosphorylated SMAD1/5/8 form heteromeric complexes with the common mediator SMAD4 before translocation into the nucleus. Thus, we next investigated whether the lack of MyD88 affected the amount of Smad4. As shown in Figures 5A,B, we found that Smad4 protein levels in nuclear extracts were significantly lower in *MyD88*^−/−^ mice compared to Wt mice, both in mice fed the standard diet and the carbonyl iron supplemented diet (Supplementary Figure 2).

**Figure 6:**
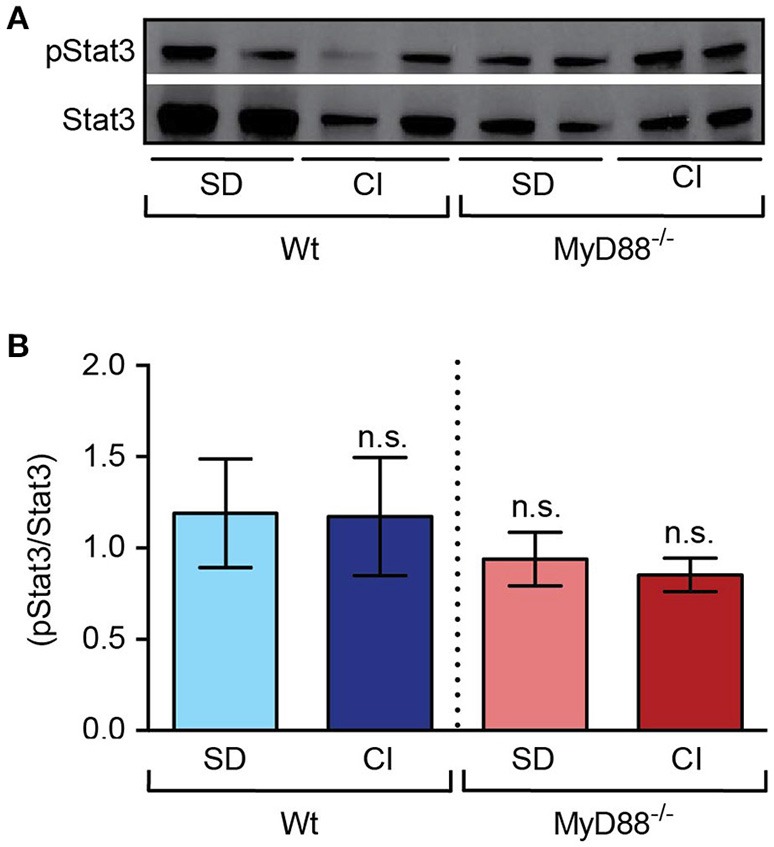
Stat3 phosphorylation in response to dietary iron-loading remains unchanged in both Wt and in *MyD88*^−/−^ mice. Liver nuclear extracts from mice fed standard diet (SD) and carbonyl iron supplemented diet (CI) were analyzed by western blots**. (A)** One representative blot probed with antibodies against phosphorylated Stat3 (pStat3) and total Stat3 (Stat3). **(B)** Graphic depicting densitometric quantification of western blots from three independent experiments. Statistical analysis was performed with one-way ANOVA: n.s., not significant compared with mice fed SD.

To exclude the influence of MyD88 mediated inflammatory response on hepcidin expression we measured Stat3 phosphorylation levels in liver nuclear extracts. As shown in Figures 6A,B, no significant differences were found regarding Stat3 phosporylation levels between mouse strains (Wt vs. *MyD88*^−/−^ mice) or treatments (standard diet vs. carbonyl iron supplemented diet).

## Discussion

Previous studies have shown that MyD88 is required for a sustained LPS-induced hypoferremic response (Layoun et al., 2012). To further study the role of MyD88 in iron sensing *in vivo*, we analyzed iron metabolism in MyD88-deficient mice. Unlike Trif-deficient mice, *MyD88*^−/−^ mice kept on a standard diet exhibited increased iron levels in the liver compared to Wt mice. Accordingly, *MyD88*^−/−^ mice also showed higher serum and liver ferritin levels. Ferritin can be abnormally elevated in a wide range of disease states including malignancy, diabetes, infection, inflammation, and chronic iron-overload syndromes (Torti and Torti, 1994; Lee and Means, 1995; Turnbull et al., 1997; Acton et al., 2006; Jacobs et al., 2009). Since *MyD88*^−/−^ mice present higher liver iron stores without activation of Stat3 phosphorylation, the increased ferritin levels in the serum most likely reflected excess iron accumulation in the liver.

High levels of iron and ferritin reflecting increased iron stores are encountered in the liver and serum of a number of disorders that are collectively called iron-loading anemias. These include thalassemia syndromes, congenital dyserythropoietic anemias, and sideroblastic anemias, all of which have the presence of robust but inefficient erythropoiesis (Nemeth and Ganz, 2006). To rule out the possibility that *MyD88*^−/−^ iron burden was due to augmented erythropoiesis activity (Papanikolaou and Pantopoulos, 2017) we measured several erythroid parameters and found no differences between *MyD88*^−/−^, Wt and Trif-deficient mice, hence ruling out a possible defect in erythropoiesis activity in *MyD88*^−/−^ mice.

Increased body iron stores are also found in the different forms of hereditary hemochromatosis (HH) (Pietrangelo, 2006). All these hereditary disorders have a common feature of inappropriately low hepcidin levels with consequential excessive iron accumulation in the liver with relative sparing of the spleen. These features are reproduced in other mouse models with hepcidin deficiency, such as *Hfe*^−/−^ and *Hjv*^−/−^ mice (Makui et al., 2005; Kent et al., 2015). Similarly, *MyD88*^−/−^ mice challenged with dietary iron were unable to appropriately control hepcidin levels, resulting in significantly higher liver iron accumulation when compared to Wt mice. These results suggest a defect in *MyD88*^−/−^ mice in the iron-sensing pathway leading to insufficient hepcidin induction. However, unlike other mouse models of iron overload, iron accumulation in the liver occurs in the absence of increased serum iron levels. A possible explanation for this difference could be enhanced ability to transport iron into hepatocytes involving heightened expression and/or activity of iron uptake molecules.

*In vivo*, hepcidin expression correlates with *Bmp6* gene expression in mice fed an iron-enriched or iron-deficient diet (Andriopoulos et al., 2009; Ryan et al., 2010). Accordingly, Bmp6-deficient mice present insufficient hepcidin levels and consequent massive iron overload (Meynard et al., 2009). Besides Bmp6, there is increasing evidence that Bmp2 also plays an important role in iron homeostasis, since mice models lacking expression of Bmp2 in liver endothelial cells develop an iron loading phenotype similar to *Bmp6*^−/−^ mice (Canali et al., 2017; Koch et al., 2017). In *MyD88*^−/−^ mice, we show that the appropriate Bmp6 and Bmp2 induction in response to iron loading is disassociated from hepcidin production. The defect was associated with the levels of Smad4 protein found in nuclear extracts from liver samples, which were diminished by about half in the *MyD88*^−/−^ mice compared to Wt mice. This finding is in line with previously reported data that described Smad4 deficiency in mice causing iron overload due to insufficient hepcidin activation (Wang et al., 2005). In addition, our results indicate that the levels of Smad5 phosphorylation were too low in *MyD88*^−/−^ mice when taking into account iron and Bmp6 levels in the liver (Kautz et al., 2008), suggesting a potential defect in Smad1/5/8 phosphorylation.

A connection between the SMAD and the MyD88 pathway has been reported that concerns the role of SMAD6 as a negative regulator of the transforming growth factor β (TGF-β) family signaling pathway, which also involves BMP (Goto et al., 2007; Lee et al., 2011). The study by Lee et al. showed SMAD6 physically interacts with MyD88. Potentially, MyD88 interactions with SMAD4 and SMAD6 may be relevant for the regulation of hepcidin, given the reported role of SMAD6 in hepcidin suppression (Lee et al., 2017).

More recently, interaction between the TLR4/MyD88 and BMP2-induced BMP/SMAD4 signaling has been demonstrated in osteoblasts, where the two pathways were found to play conflicting roles in the regulation of BMP-2-induced osteoblast differentiation (Huang et al., 2013). In their study, Huang et al. showed that LPS-mediated inflammatory environment inhibits BMP-2-induced osteogenic differentiation through the crosstalk between TLR4/MyD88/NF-κB and BMP/SMAD signaling, which contrasts with our present findings. However, cross-talk between the inflammatory and SMAD pathways could be cell-context specific, since receptor expression seems to modulate the specificity of TGF-β signaling pathways (Murakami et al., 2009). In fact, stimulation of SMAD1/5/8 signaling and hepcidin induction during inflammation by other TGF-β superfamily members, such as activin B (Besson-Fournier et al., 2012), has been shown to be unique in liver cells (Canali et al., 2016). The exact nature of the cross-talk between MyD88 and SMAD pathways remains to be defined, and further studies are needed to understand whether MyD88 may, for example, stabilize SMAD4 and/or have a role in the translocation of the SMAD4/phosphorylated SMAD1/5/8 complexes into the nucleus.

In summary, we report here for the first time that MyD88 is critical for appropriate nuclear Smad4 protein expression, Smad1/5/8 phosphorylation, and for the induction of hepcidin upon challenge with dietary iron. Together, our data identify a new role for MyD88 as a potential key molecule in the BMP signaling pathway mediated by SMAD proteins.

## Author contributions

MS-M, AL, GF, and AC contributed to the investigation, validation, methodology, and formal analysis. MS-M, AL, and MS additionally contributed to the conceptualization and writing of the original draft of the manuscript. MS additionally contributed to the visualization, supervision, and funding acquisition of the study.

### Conflict of interest statement

The authors declare that the research was conducted in the absence of any commercial or financial relationships that could be construed as a potential conflict of interest.
