# Supplementary material for: MyD88 Adaptor Protein Is Required for Appropriate Hepcidin Induction in Response to Dietary Iron Overload in Mice
Source: Front Physiol. 2018 Mar 5;9:159. doi: 10.3389/fphys.2018.00159 (PMC5845127; doi:10.3389/fphys.2018.00159)

Supplementary Figure 1:

Liver *Bmp2* mRNA levels. Wt and MyD88$^{-/-}$ mice were fed a standard diet (SD) or carbonyl iron supplemented diet (CI) for two weeks. *Bmp2* mRNA levels in the liver. The results are representative of three independent experiments using n = 4-8 mice per group in each experiment. Statistical analysis was performed with one-way ANOVA: n.s. = not significant compared with Wt mice fed the same diet.

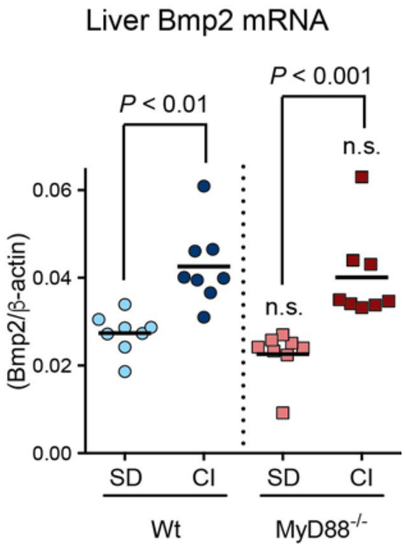

Supplement: Supplementary file 1 [file Image1.pdf]
