# Supplementary material for: MyD88 Adaptor Protein Is Required for Appropriate Hepcidin Induction in Response to Dietary Iron Overload in Mice
Source: Front Physiol. 2018 Mar 5;9:159. doi: 10.3389/fphys.2018.00159 (PMC5845127; doi:10.3389/fphys.2018.00159)

Supplementary Figure 2:

Liver Smad4 protein levels. Liver nuclear extracts from mice fed (A) standard diet; and (B) carbonyl iron supplemented diet were analyzed by western blots. One representative blot is shown probed with an antibody against Smad4. Blots were stripped and reprobed with an antibody to β-actin.

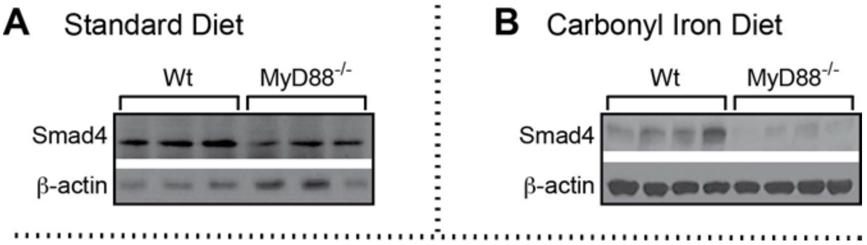

Supplement: Supplementary file 2 [file Image2.pdf]
